# Production of Methyl-Iodide in the Environment

**DOI:** 10.3389/fmicb.2021.804081

**Published:** 2021-12-23

**Authors:** Eva Duborská, Katarína Balíková, Michaela Matulová, Ondřej Zvěřina, Bence Farkas, Pavol Littera, Martin Urík

**Affiliations:** ^1^Faculty of Natural Sciences, Institute of Laboratory Research on Geomaterials, Comenius University in Bratislava, Bratislava, Slovakia; ^2^Department of Public Health, Faculty of Medicine, Masaryk University, Brno, Czechia

**Keywords:** biomethylation, iodine, biogenic methyl-iodide, volatilization, iodide

## Abstract

Iodine is an essential micronutrient for most of the living beings, including humans. Besides its indispensable role in animals, it also plays an important role in the environment. It undergoes several chemical and biological transformations resulting in the production of volatile methylated iodides, which play a key role in the iodine’s global geochemical cycle. Since it can also mitigate the process of climate change, it is reasonable to study its biogeochemistry. Therefore, the aim of this review is to provide information on its origin, global fluxes and mechanisms of production in the environment.

## Introduction

Atmospheric iodine species, such as methyl-iodides, are distributed to terrestrial and aquatic environments *via* wet and dry depositions. The primary processes that affect iodine’s allocation and speciation in the atmosphere are photochemical reactions (Moore and Zafiriou, 1994), and the production of volatile iodide’s transformants by marine phytoplankton, cyanobacteria and algae, which are the main sources of biogenic alkyl-iodides in the atmosphere (Carpenter, 2003).

The environmental relevance of the methyl-iodide is highlighted by the estimates which suggest that in the regions between 40 N° and 40 S° up to 174 Gg of methyl-iodide is produced annually *via* biological and photochemical processes (Stemmler et al., 2014). Furthermore, according to Bell et al. (2002), almost 70% of the annual flux of methyl-iodide originates from the open oceanic waters. These accrued volatile organo-iodine species participate in the formation of cloud condensation nuclei (Saunders and Saiz-Lopez, 2009), which can cause albedo and regional cooling with a negative effect on climate. Furthermore, the reactive methyl-iodide species also take part in the depletion of ozone layer (Chameides and Davis, 1980; Solomon et al., 1994; Davis et al., 1996).

## Mechanisms of Methyl-Iodides’ Genesis

Methyl-iodide compounds are produced in seawater *via* the reaction between photochemically produced methyl and iodine radicals, typically in temperate and tropical waters (Moore and Zafiriou, 1994). Nevertheless, the illumination, as well as the acidic pH, and the dissolved organic carbon presence accelerate the production of methyl-iodide in sea water (Chen et al., 2020).

Besides photochemical reaction, iodide methylation can be mediated biologically. Thayer (2002) noted that there are three pathways of iodine methylation. The first pathway is the reaction of iodide with β-dimethylsulphoniopropionate (DMSP), an algal osmoprotectant. However, the production of methyl-halides through this reaction is not favored, because of its endothermic character. Thus, it was estimated that only less than 1% of methyl-halides in the atmosphere are produced by this reaction (Hu and Moore, 1996).

The second mechanism suggests the direct transformation of iodide into methylated species *via* enzymatic transformation originally proposed for arsenic methylation (Challenger, 1951). *Via* this methylation pathway, the transformation of inorganic iodide into methylated compounds involves the nucleophilic attack of iodide at the electrophilic CH_3_^+^S site of the S-adenosyl-L-methionine methyl donor mediated by the methyltransferases (Itoh et al., 2009). This pathway is proved to be favored by various microorganism. Amachi et al. (2001) demonstrated that methyl-iodide producing ability is widespread among marine and terrestrial bacteria (e.g., *Variovorax* sp., *Photobacterium leiognathi*), while anaerobic microorganisms (e.g., methanogens) produce methyl-iodide less likely. In plants, Hol-1 protein was identified as a methyltransferase that catalyzes the S-adenosyl-L-methionine dependent methylation of halides (Landini et al., 2012).

The third mechanism suggests the formation of methyl-iodide by vanadium-dependent haloperoxidases (Punitha et al., 2018) in the presence of hydrogen peroxide (Leblanc et al., 2006). Iodine specific iodoperoxidase was identified in various marine diatoms and macrophytes (Colin et al., 2003). Such peroxidase is also considered to be responsible for iodine methylation in plants (Neidleman and Geigert, 1987).

## Production of Methyl-Iodide in Seawater

Elemental iodine is also released into the atmosphere by photochemical oxidation of iodide by UV radiation in the presence of O_2_ (Saunders and Saiz-Lopez, 2009):


(1) $$4I^{-}+{\text{O}}_{2}+{\text{H}}_{2}\text{O}+hv\to 2I_{2}+2{\mathrm{OH}}^{-}$$

Methylated iodine compounds are produced photochemically in seawater *via* the reaction between photochemically produced methyl radicals and iodine atoms (Moore and Zafiriou, 1994) *via* the reaction:


(2) $${\text{CH}}_{3}^{.}+\cdot \text{I}\to {\text{CH}}_{3}\text{I}$$

While iodine radicals can be produced *via* the reaction of I_2_ with NO_3_ radicals while sunlight is not needed for this reaction (Saiz-Lopez et al., 2006):


(3) $${\text{I}}_{2}+\cdot {\text{NO}}_{3}\to {\mathrm{INO}}_{3}+\text{I}\cdot$$

Other possible pathways are suggested too by Moore and Zafiriou (1994) but they are unlikely to occur in natural non-contaminated waters.

## Production of Methyl-Iodide by Marine Algae

Küpper et al. (2018) studied iodine metabolism in the filamentous brown alga *Ectocarpus siliculosus*, whose predominant emission was methyl-iodide among various other volatile halogenated compounds. Elevated methyl-iodide concentrations were also found in the algal beds of *Laminaria digitata* at the coasts of Scotland (Nightingale et al., 1995) and California (Manley et al., 1992). Excessive amounts of methyl-iodide levels are also common in the beds of giant kelp (*Macrocystis pyrifera*), but it is not considered a significant source of iodine (Manley and Dastoor, 1987). Large amounts of iodine were observed also above the sea ice in the Weddell sea at Antarctic coast presumably originating from diatoms found in the sea ice (Atkinson et al., 2012).

Since algal activity is sensitive to light, it should also affect the production of methyl-iodide. However, the results are contradicting. While Nightingale et al. (1995) indicated that the methyl-iodide production by *L*. *digitata* was enhanced in the dark, Carpenter et al. (2000) noted that the organoiodine production by macroalgae is stimulated by light.

## Production of Methyl-Iodide by Bacteria

Aerobic incubation of seawater showed that the iodine is volatilized from the seawater biologically. Further investigation showed that the production of methyl-iodide is due to bacterial activity, thus, the addition of antibiotics caused a significant decrease in iodine’s volatilization (Amachi et al., 2004). Fuse et al. (2003) isolated two strains of iodine-producing bacteria, identified as *Roseovarius* spp., which were capable of producing not only methyl-iodide but also other methyl-iodide derivatives from the iodide supplemented media. Members of the *Proteobacteria*, *Cytophaga-Flexibacter-Bacteroides* group (Amachi et al., 2004), and also the strains of *Alteromonas macleodii* and *Vibrio splendidus* (Amachi et al., 2001) can also facilitate organo-iodide release from the seawater. Smythe-Wright et al. (2006) estimated that the methyl-iodide production by cyanobacteria *Prochlorococcus* at 5.3 × 10^11^ g year^–1^. However, Brownell et al. (2010) suggested, that methyl iodide production of this species accounts only about 0.3% of the global marine production, in future it may represent a remarkable source of volatile iodine species in low latitude waters of the Atlantic and Indian oceans, since the recent models showed an increase in the abundance of this bacterial species by up to 15% due to global warming (Smythe-Wright et al., 2006) while the production rate is dependent on the physiological state of cells and culture conditions (Hughes et al., 2011). Under optimal conditions, some bacterial strains belonging to *Erythobacter* are capable of producing as much as 49 pmol L^–1^ h^–1^ of methyl-iodide (Fujimori et al., 2012). Nevertheless, Gómez-Consarnau et al. (2021) noted that the methyl-iodide synthesis is a generalized process in representatives of the major marine heterotrophic bacterial groups.

## Production of Methyl-Iodide in Terrestrial Environments

In soils, the presence of manganese oxides can contribute to the formation of methyl-iodide abiotically. Under conditions that the iodide and the natural organic matter is present, Allard et al. (2010) showed that the manganese sand can initiate the formation of methyl-iodide. However, the speciation of the final product depends on the properties of the present organic matter. While the methyl-iodide was produced in the presence of humic acids, the abiotic synthesis of high-molecular alkyl-iodides, such as propyl- and butyl-iodides, was enhanced when the other major fractions of soil organic matter were also present and exposed to iodine.

The iodine volatilization from soils is stimulated by plants, especially in flooded soils, partially due to the action of enzymes produced by the roots (Muramatsu and Yoshida, 1995). Furthermore, in flooded soils where redox potential is low, thus, under such conditions, iodine is reductively desorbed from the soil particles to soil solution as iodide and transformed into reactive volatile species (Yuita, 1992). Some soil organic compounds with large carbonyl moieties, or alkyl-chlorides can enhance the formation of volatile iodine compounds, while aromatic compounds can stabilize the iodine in soil (Taghipour and Evans, 2001). Biotic transformation of iodine into the methyl-iodides plays also prominent role in the oligotrophic sediments at nuclear waste sites where the isolated microcosm possessed iodine methylation abilities (Bagwell et al., 2019).

### Production of Methyl-Iodide by Plants

Methyl-halides do not play a fundamental role in the plant development (Rhew et al., 2003), although iodine has reportedly adverse effects on plant’s growth at high concentrations (Duborská et al., 2018). Therefore, the accumulated iodine is preferentially volatilized as methyl-iodide through the activity of specific methyl transferase encoded by *HOL* (*HARMLESS TO OZONE LAYER*) genes (Carlessi et al., 2021). There are two types of *HOL* genes from which the *HOL-1* is responsible for iodine methylation (Landini et al., 2012).

Contribution of methyl-iodide production by higher plants on total flux of iodine to atmosphere from soils is minor on global scale, however, it was estimated that the total annual amount of volatilized iodine from rice fields approximates 2 × 10^10^ g (Muramatsu and Yoshida, 1995). Furthermore, the emission of methyl-iodide from the shoots of rice plant are more significant in comparison to the contribution of flooded soil surface (Muramatsu and Yoshida, 1995).

The enzyme responsible for iodide methylation in leaves of cabbage (*Brassica oleracea*) was classified as a halide/bisulfide methyltransferase with pH optimum for iodide methylation in the range of 5.5–7. It catalyzes the S-adenosyl-L-methionine-dependent methylation of the halides (iodide, bromide, and chloride) to monohalogenated methanes (Attieh et al., 1995).

Various other species of *Brassicaceae* family (e.g., *B*. *rapa* and *Raphanus sativus*) and species of the *Poaceae* family, such as *Triticum aestivum*, were also reported to be able of releasing of volatile iodine species (Rhew et al., 2003); and it is generally accepted that the iodide methylation is widespread among higher plants (Saini et al., 1995).

### Production of Methyl-Iodide by Fungi

Generally, fungi are not considered relevant producers of methylated iodine species on global scale. However, considering their abundance in the environment, more attention should be taken toward their investigation. So far, the methyl-iodide was the only reported volatile organic transformant of iodine produced by fungi (Ban-Nai et al., 2006). The release of environmentally significant amount of methyl-iodide was reported from wood-rotting fungus *Phellinus pomaceus* (Harper, 1985). Various Basidiomycota species, including *Lentinula edodes*, *Hebeloma vinosophyllum*, *Pleurotus ostreatus* and *Agaricus bisporus* (Ban-Nai et al., 2006), as well as the ectomycorrhizal fungal species *Cenococcum geophilum*, *Hebeloma crustuliniforme*, *Inocybe maculata*, and *Laccaria laccata* (Redeker et al., 2004) were also reported to produce methyl-iodide under laboratory conditions. However, the exact mechanism of iodine biovolatilization by fungi is not yet sufficiently explained; and, thus, it was hypothesized that both intracellular and extracellular transformations into methyl-iodides may occur (Urík et al., 2007; Duborská et al., 2017). It is also very likely that the filamentous fungi can facilitate iodine methylation indirectly *via* production of extracellular metabolites, e.g., the strains of *Alternaria alternata*, *Fusarium oxysporum*, *Penicillium roqueforti*, *P*. *chrysogenum*, *Cladosporium cladosporioides*, *Aspergillus niger* and *A*. *oryzae*, which possess the abilities of methyl-iodide production. While the precursor for such transformation by fungi in aquatic environments was reportedly almost exclusively iodide, the preferable source in terrestrial systems seems to be iodate (Duborská et al., 2017).

## Global Effluxes of Methyl-Iodides

While the global annual ocean-atmosphere flux of methyl-iodide is estimated at 219 Gg, its local estimations vary significantly depending on the region or season. In seawater, the photochemical and biological processes are co-occurring, thus, both biological and photochemical production pathways (Table 1) need to be taken into account to approximate the global production of methyl-iodide (Stemmler et al., 2014).

**TABLE 1:** Global production, loss, emission, and inventory of methyl-iodide (Stemmler et al., 2014).

Production pathway	
Production (Gg.year^–1^)	348.27
Biological (%)	0.2
Photochemical^a^ (%)	28
Photochemical^b^ (%)	72
Net emission (Gg.year^–1^)	170.61
Loss (Gg.year^–1^)	164.97
Inventory (Gg)	14.10

*^a^Production from semi-labile dissolved organic carbon. ^b^Production from refractory dissolved organic carbon.*

While the biological production of methyl-iodide is observable during the whole year in the tropical eastern Atlantic and equatorial Pacific zones, the fluxes in regions with boreal winter occur predominantly from the atmosphere to the ocean (Stemmler et al., 2014). In the southern ocean, the biological production is limited to the period of austral spring (Stemmler et al., 2014). On the other hand, a large number of macroalgae isolated from the Antarctic are capable of methyl-iodide production (Laturnus et al., 1998). Smythe-Wright et al. (2006) estimated that the annual flux of methyl-iodide produced by cyanobacterium *Prochlorococcus* alone is 4.3 × 10^9^mol. However, Manley and de la Cuesta (1997) stated that methyl-iodide produced by the marine phytoplankton is not significant on global scale. Butler et al. (1981) suggested that the methyl-iodide produced by the phytoplankton is only a short-lived intermediate which is converted to methyl-chloride.

Nevertheless, the terrestrial sources are not as significant as the marine sources. However, the rice plants possessing the ability to methylate iodide are considered a significant source with 5% contribution to the global flux of methyl-iodide (Redeker et al., 2000). Furthermore, Dimmer et al. (2001) reported annual flux of 1.4 Gg from peatland ecosystems and 7.3 Gg from wetlands. Burning of biomass can also contribute to the annual flux of methyl-iodide by up to 3.5 Gg (Blake et al., 1996).

## Conclusion

Since methyl-iodides play an important role in the depletion of ozone layer and the formation of cloud condensation nuclei which can results in regional cooling with a negative climate feedback, the release of organoiodine compounds can mitigate global warming, causing a further increase in number of iodide-methylating bacteria and algae. This short review provides a biogeochemical context for such a scenario since it notes the mechanisms that are behind the formation and propagation of methyl-iodide into the environment. It also highlights that process of transforming iodine into methyl-iodine is regulated and accelerated biotically, and this ability is almost universal across the genera of various organisms, including phytoplankton, fungi and plants as well. To conclude, the research to improve understanding of the contribution of biogenic sources on methyl-iodide distribution and their impact on the environment is much needed.

## Author Contributions

ED and MU: writing—original draft preparation. MM, OZ, KB, and BF: writing—review and editing. PL: supervision. MU: funding acquisition. All authors have read and agreed to the published version of the manuscript.

## Conflict of Interest

The authors declare that the research was conducted in the absence of any commercial or financial relationships that could be construed as a potential conflict of interest.

## Publisher’s Note

All claims expressed in this article are solely those of the authors and do not necessarily represent those of their affiliated organizations, or those of the publisher, the editors and the reviewers. Any product that may be evaluated in this article, or claim that may be made by its manufacturer, is not guaranteed or endorsed by the publisher.

## References

[B1] Allard S. Gallard H. Fontaine C. Croué J.-P. (2010). Formation of methyl iodide on a natural manganese oxide. *Water Res.* 44 4623–4629. 10.1016/j.watres.2010.06.008 20580399

[B2] Amachi S. Kamagata Y. Kanagawa T. Muramatsu Y. (2001). Bacteria mediate methylation of iodine in marine and terrestrial environments. *Appl. Environ. Microbiol.* 67 2718–2722. 10.1128/AEM.67.6.2718-2722.2001 11375186 PMC92930

[B3] Amachi S. Kasahara M. Fujii T. Shinoyama H. Hanada S. Kamagata Y. (2004). Radiotracer experiments on biological volatilization of organic iodine from coastal seawaters. *Geomicrobiol. J.* 21 481–488.

[B4] Atkinson H. M. Huang R. J. Chance R. Roscoe H. K. Hughes C. Davison B. (2012). Iodine emissions from the sea ice of the Weddell Sea. *Atmos. Chem. Phys.* 12 11229–11244.

[B5] Attieh J. M. Hanson A. D. Saini H. S. (1995). Purification and characterization of a novel methyltransferase responsible for biosynthesis of halomethanes and methanethiol in *Brassica oleracea*. *J. Biol. Chem.* 270 9250–9257. 10.1074/jbc.270.16.9250 7721844

[B6] Bagwell C. E. Zhong L. Wells J. R. Mitroshkov A. V. Qafoku N. P. (2019). Microbial methylation of iodide in unconfined aquifer sediments at the Hanford Site, USA. *Front. Microbiol.* 10:2460. 10.3389/fmicb.2019.02460 31708909 PMC6821650

[B7] Ban-Nai T. Muramatsu Y. Amachi S. (2006). Rate of iodine volatilization and accumulation by filamentous fungi through laboratory cultures. *Chemosphere* 65 2216–2222. 10.1016/j.chemosphere.2006.05.047 16828143

[B8] Bell N. Hsu L. Jacob D. J. Schultz M. G. Blake D. R. Butler J. H. (2002). Methyl iodide: atmospheric budget and use as a tracer of marine convection in global models. *J. Geophys. Res.* 107:4340.

[B9] Blake N. J. Blake D. R. Sive B. C. Chen T.-Y. Rowland F. S. Collins J. E. Jr. (1996). Biomass burning emissions and vertical distribution of atmospheric methyl halides and other reduced carbon gases in the South Atlantic region. *J. Geophys. Res. Atmos.* 101 24151–24164. 10.1029/96jd00561

[B10] Brownell D. K. Moore R. M. Cullen J. J. (2010). Production of methyl halides by *Prochlorococcus* and *Synechococcus*. *Glob. Biogeochem. Cycles* 24:GB2002.

[B11] Butler E. C. V. Smith J. D. Fisher N. S. (1981). Influence of phytoplankton on iodine speciation in seawater. *Limnol. Oceanogr.* 26 382–386.

[B12] Carlessi M. Mariotti L. Giaume F. Fornara F. Perata P. Gonzali S. (2021). Targeted knockout of the gene OsHOL1 removes methyl iodide emissions from rice plants. *Sci. Rep.* 11:17010. 10.1038/s41598-021-95198-x 34426588 PMC8382704

[B13] Carpenter L. J. (2003). Iodine in the marine boundary layer. *Chem. Rev.* 103 4953–4962. 10.1021/cr0206465 14664638

[B14] Carpenter L. J. Malin G. Liss P. S. Küpper F. C. (2000). Novel biogenic iodine-containing trihalomethanes and other short-lived halocarbons in the coastal east Atlantic. *Glob. Biogeochem. Cycles* 14 1191–1204. 10.1029/2000gb001257

[B15] Challenger F. (1951). “Biological methylation,” in *Advances in Enzymology and Related Areas of Molecular Biology*, ed. Purich D. (Hoboken, NJ: Wiley-Interscience), 429–491.

[B16] Chameides W. L. Davis D. D. (1980). Iodine: its possible role in tropospheric photochemistry. *J. Geophys. Res. Oceans* 85 7383–7398. 10.1029/jc085ic12p07383

[B17] Chen Y. Liu S. Yang G. He Z. (2020). Influence factors on photochemical production of methyl iodide in seawater. *J. Ocean Univ. China* 19 1353–1361. 10.1007/s11802-020-4463-8

[B18] Colin C. Leblanc C. Wagner E. Delage L. Leize-Wagner E. Van Dorsselaer A. (2003). The brown algal kelp *Laminaria digitata* features distinct bromoperoxidase and iodoperoxidase activities*. *J. Biol. Chem.* 278 23545–23552. 10.1074/jbc.M300247200 12697758

[B19] Davis D. Crawford J. Liu S. Mckeen S. Bandy A. Thornton D. (1996). Potential impact of iodine on tropospheric levels of ozone and other critical oxidants. *J. Geophys. Res. Atmos.* 101 2135–2147. 10.1029/95jd02727

[B20] Dimmer C. H. Simmonds P. G. Nickless G. Bassford M. R. (2001). Biogenic fluxes of halomethanes from Irish peatland ecosystems. *Atmos. Environ.* 35 321–330. 10.1016/s1352-2310(00)00151-5

[B21] Duborská E. Urík M. Bujdoš M. (2017). Comparison of iodide and iodate accumulation and volatilization by filamentous fungi during static cultivation. *Water Air Soil Pollut.* 228:225.

[B22] Duborská E. Urík M. Kubová J. (2018). Interaction with soil enhances the toxic effect of iodide and iodate on barley (*Hordeum vulgare* L.) compared to artificial culture media during initial growth stage. *Arch. Agron. Soil Sci.* 64 46–57. 10.1080/03650340.2017.1328104

[B23] Fujimori T. Yoneyama Y. Taniai G. Kurihara M. Tamegai H. Hashimoto S. (2012). Methyl halide production by cultures of marine *proteobacteria* erythrobacter and *pseudomonas* and isolated bacteria from brackish water. *Limnol. Oceanogr.* 57 154–162. 10.4319/lo.2012.57.1.0154

[B24] Fuse H. Inoue H. Murakami K. Takimura O. Yamaoka Y. (2003). Production of free and organic iodine by Roseovarius spp. *FEMS Microbiol. Lett.* 229 189–194. 10.1016/S0378-1097(03)00839-5 14680698

[B25] Gómez-Consarnau L. Klein N. J. Cutter L. S. Sañudo-Wilhelmy S. A. (2021). Growth rate-dependent synthesis of halomethanes in marine heterotrophic bacteria and its implications for the ozone layer recovery. *Environ. Microbiol. Rep.* 13 77–85. 10.1111/1758-2229.12905 33185965

[B26] Harper D. B. (1985). Halomethane from halide ion—a highly efficient fungal conversion of environmental significance. *Nature* 315 55–57.

[B27] Hu Z. Moore R. M. (1996). Kinetics of methyl halide production by reaction of DMSP with halide ion. *Mar. Chem.* 52 147–155. 10.1016/0304-4203(95)00077-1

[B28] Hughes C. Franklin D. J. Malin G. (2011). Iodomethane production by two important marine cyanobacteria: *Prochlorococcus marinus* (CCMP 2389) and *Synechococcus* sp. (CCMP 2370). *Mar. Chem.* 125 19–25. 10.1016/j.marchem.2011.01.007

[B29] Itoh N. Toda H. Matsuda M. Negishi T. Taniguchi T. Ohsawa N. (2009). Involvement of S-adenosylmethionine-dependent halide/thiol methyltransferase (HTMT) in methyl halide emissions from agricultural plants: isolation and characterization of an HTMT-coding gene from *Raphanus sativus*(daikon radish). *BMC Plant Biol.* 9:116. 10.1186/1471-2229-9-116 19723322 PMC2752461

[B30] Küpper F. C. Miller E. P. Andrews S. J. Hughes C. Carpenter L. J. Meyer-Klaucke W. (2018). Emission of volatile halogenated compounds, speciation and localization of bromine and iodine in the brown algal genome model *Ectocarpus siliculosus*. *J. Biol. Inorgan. Chem.* 23 1119–1128. 10.1007/s00775-018-1539-7 29523971

[B31] Landini M. Gonzali S. Kiferle C. Tonacchera M. Agretti P. Dimida A. (2012). Metabolic engineering of the iodine content in *Arabidopsis*. *Sci. Rep.* 2:338. 10.1038/srep00338 PMC3313481 22468225

[B32] Laturnus F. Adams F. C. Wiencke C. (1998). Methyl halides from Antarctic macroalgae. *Geophys. Res. Lett.* 25 773–776. 10.1029/98gl00490

[B33] Leblanc C. Colin C. Cosse A. Delage L. La Barre S. Morin P. (2006). Iodine transfers in the coastal marine environment: the key role of brown algae and of their vanadium-dependent haloperoxidases. *Biochimie* 88 1773–1785. 10.1016/j.biochi.2006.09.001 17007992

[B34] Manley S. L. Dastoor M. N. (1987). Methyl halide (CH3X) production from the giant kelp, *Macrocystis*, and estimates of global CH3X production by kelp1. *Limnol. Oceanogr.* 32 709–715. 10.4319/lo.1987.32.3.0709

[B35] Manley S. L. de la Cuesta J. L. (1997). Methyl iodide production from marine phytoplankton cultures. *Limnol. Oceanogr.* 42 142–147. 10.4319/lo.1997.42.1.0142

[B36] Manley S. Goodwin K. North W. (1992). Laboratory production of bromoform, methylene bromide, and methyl iodide by macroalgae and distribution in nearshore southern California waters. *Limnol. Oceanogr.* 37 1652–1659. 10.4319/lo.1992.37.8.1652

[B37] Moore R. M. Zafiriou O. C. (1994). Photochemical production of methyl iodide in seawater. *J. Geophys. Res.* 99 16415–16420. 10.1016/j.envpol.2021.116749 33639487

[B38] Muramatsu Y. Yoshida S. (1995). Volatilization of methyl iodide from the soil-plant system. *Atmos. Environ.* 29 21–25. 10.1016/1352-2310(94)00220-f

[B39] Neidleman S. L. Geigert J. (1987). Biological halogenation: roles in nature, potential in industry. *Endeavour* 11 5–15. 10.1016/0160-9327(87)90163-3 2436889

[B40] Nightingale P. D. Malin G. Liss P. S. (1995). Production of chloroform and other low molecular-weight halocarbons by some species of macroalgae. *Limnol. Oceanogr.* 40 680–689. 10.4319/lo.1995.40.4.0680

[B41] Punitha T. Phang S. M. Juan J. C. Beardall J. (2018). Environmental control of vanadium haloperoxidases and halocarbon emissions in macroalgae. *Mar. Biotechnol. (N. Y.)* 20 282–303. 10.1007/s10126-018-9820-x 29691674

[B42] Redeker K. R. Treseder K. K. Allen M. F. (2004). Ectomycorrhizal fungi: a new source of atmospheric methyl halides? *Glob. Change Biol.* 10 1009–1016. 10.1111/j.1529-8817.2003.00782.x

[B43] Redeker K. R. Wang N. Low J. C. Mcmillan A. Tyler S. C. Cicerone R. J. (2000). Emissions of methyl halides and methane from rice paddies. *Science* 290 966–969. 10.1126/science.290.5493.966 11062125

[B44] Rhew R. C. Ostergaard L. Saltzman E. S. Yanofsky M. F. (2003). Genetic control of methyl halide production in *Arabidopsis*. *Curr. Biol.* 13 1809–1813. 10.1016/j.cub.2003.09.055 14561407

[B45] Saini H. S. Attieh J. M. Hanson A. D. (1995). Biosynthesis of halomethanes and methanethiol by higher plants *via* a novel methyltransferase reaction. *Plant Cell Environ.* 18 1027–1033. 10.1111/j.1365-3040.1995.tb00613.x

[B46] Saiz-Lopez A. Plane J. M. C. Mcfiggans G. Williams P. I. Ball S. M. Bitter M. (2006). Modelling molecular iodine emissions in a coastal marine environment: the link to new particle formation. *Atmos. Chem. Phys.* 6 883–895.

[B47] Saunders R. W. Saiz-Lopez A. (2009). “Iodine in the air: origin, transformation, and exchange to mammals,” in *Comprehensive Handbook of Iodine*, eds Preedy V. R. Burrow G. N. Watson R. (San Diego, CA: Academic Press), 73–82.

[B48] Smythe-Wright D. Boswell S. M. Breithaupt P. Davidson R. D. Dimmer C. H. Eiras Diaz L. B. (2006). Methyl iodide production in the ocean: implications for climate change. *Glob. Biogeochem. Cycles* 20:GB3003.

[B49] Solomon S. Garcia R. R. Ravishankara A. (1994). On the role of iodine in ozone depletion. *J. Geophys. Res. Atmos.* 99 20491–20499. 10.1029/94jd02028

[B50] Stemmler I. Hense I. Quack B. Maier-Reimer E. (2014). Methyl iodide production in the open ocean. *Biogeosciences* 11 4459–4476. 10.5194/bg-11-4459-2014

[B51] Taghipour F. Evans G. J. (2001). Radioiodine volatilization in the presence of organic compounds. *Nuclear Technol.* 134 208–220.

[B52] Thayer J. S. (2002). Biological methylation of less-studied elements. *Appl. Organometal. Chem.* 16 677–691. 10.1002/aoc.375

[B53] Urík M. Čerňanský S. Ševc J. Šimonovičová A. Littera P. (2007). Biovolatilization of arsenic by different fungal strains. *Water Air Soil Pollut.* 186 337–342. 10.1007/s11270-007-9489-7

[B54] Yuita K. (1992). Dynamics of iodine, bromine, and chlorine in soil .2. Chemical forms of iodine in soil solutions. *Soil Sci. Plant Nutr.* 38 281–287.

